# Supplementary material for: Distinct patterns of volcano deformation for hot and cold magmatic systems
Source: Nat Commun. 2025 Jan 9;16:532. doi: 10.1038/s41467-024-55443-z (PMC11718178; doi:10.1038/s41467-024-55443-z)
Supplement: Supplementary file 1 — Supplementary Information [file 41467_2024_55443_MOESM1_ESM.pdf]

Supplementary Information for

Distinct patterns of volcano deformation for hot and cold magmatic systems

Gregor Weber[1], Juliet Biggs[1], and Catherine Annen[2]

[1] COMET, School of Earth Sciences, University of Bristol, Bristol, United Kingdom.

[2] Institute of Geophysics of the Czech Academy of Sciences, Prague, Czech Republic.

Corresponding author: Gregor Weber (gregor_weber@bristol.ac.uk)

Contents of this file

Figures S1 to S7
Table S1, S2

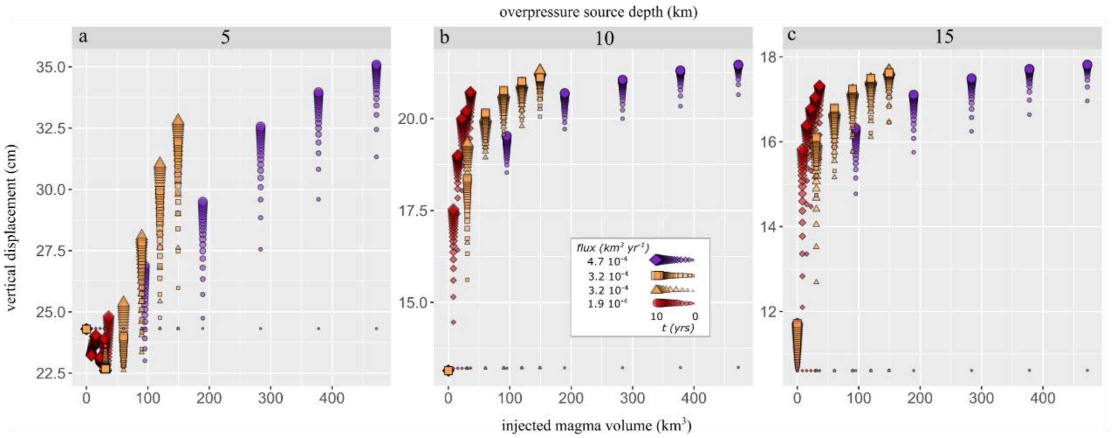

Figure S1. Total volume of injected magma during long-term magmatism (km^3) versus vertical displacement. The different colored symbols represent different magma fluxes. Purple circles are drawn for the high magma flux scenario, orange squares for the intermediate flux, and red diamonds for the low flux. The orange triangles represent the results of a repeat run with the same flux as the intermediate scenario but with different randomization of dike injection locations. Symbol size corresponds to time in years. a) Results for an overpressure source of 5 km, b) for 10 km, and 3) for a 15 km deep source. Note the difference in scale of the Y-axis.

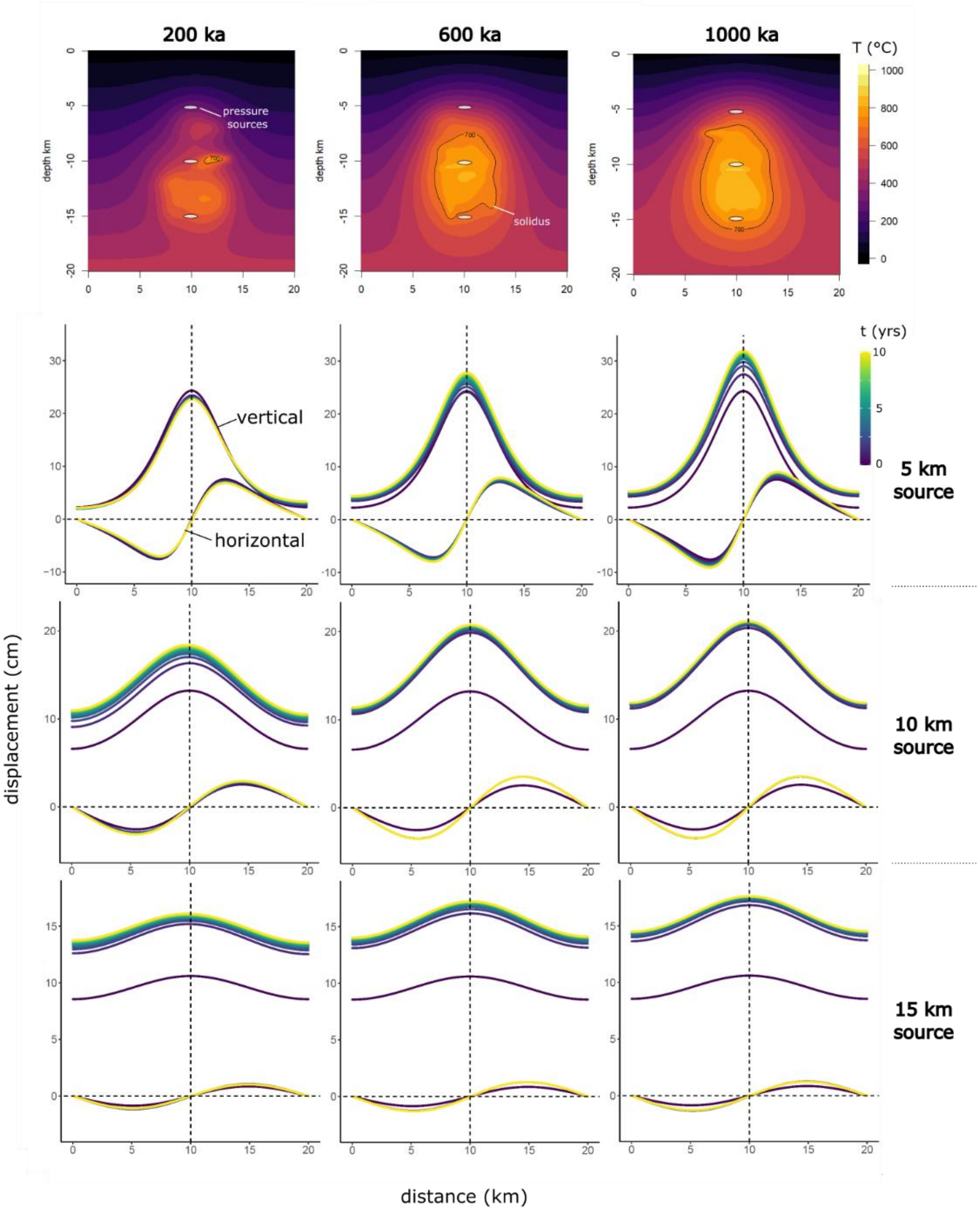

Figure S2. Impact of long-term magma systems evolution on spatial surface deformation pattern. 2D plots display the thermal state of the crust after 200, 600, and 1000 ka of magma injection (intermediate flux scenario), with white ellipsoids marking the location of overpressure sources. The black line represents the solidus temperature of 700℃. Each column shows the vertical and horizontal component of surface displacement (cm) versus distance (km) beneath the respective temperature field for overpressure sources at 5, 10 or 15 km depth. Horizontal displacements are either negative or positive, depending on the direction from the centre. Color coding of the curves reflects the time evolution in years of the visco-elastic phase.

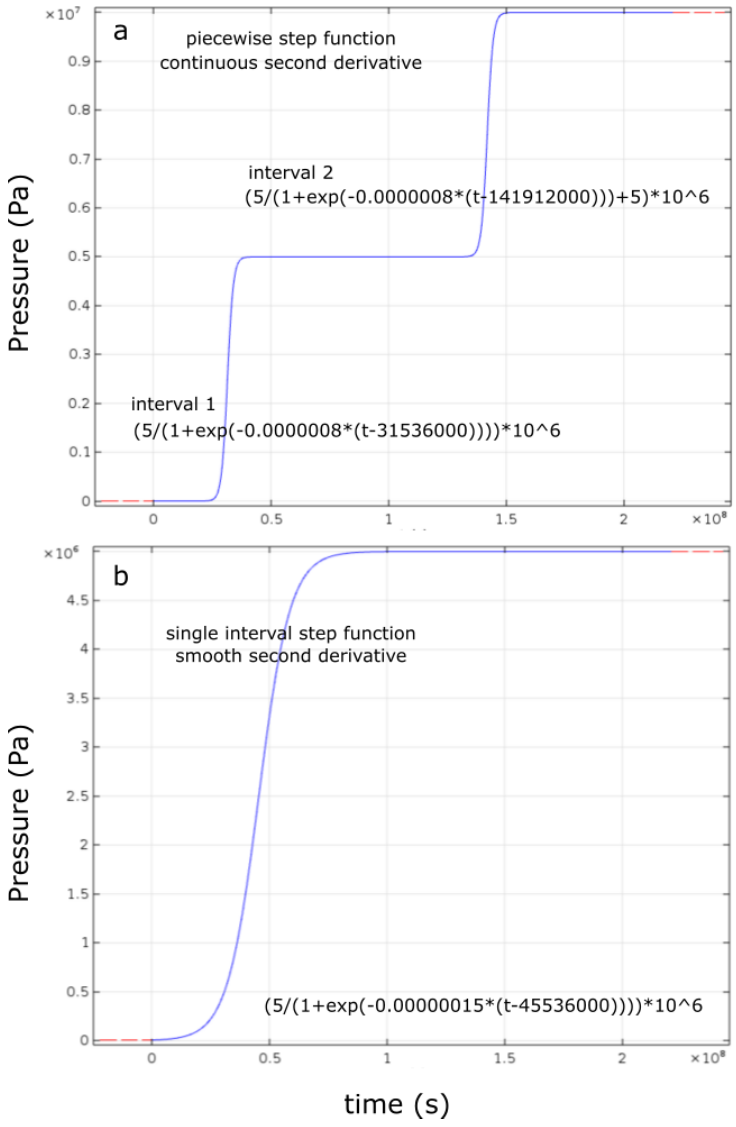

Figure S3. Overpressure (Pa) with time (s) used for model calculations in Fig. 8. a) Piecewise defined smooth step function with two intervals. b) Single interval step function with smooth second derivative. Red dashed lines indicate constant value extrapolation.

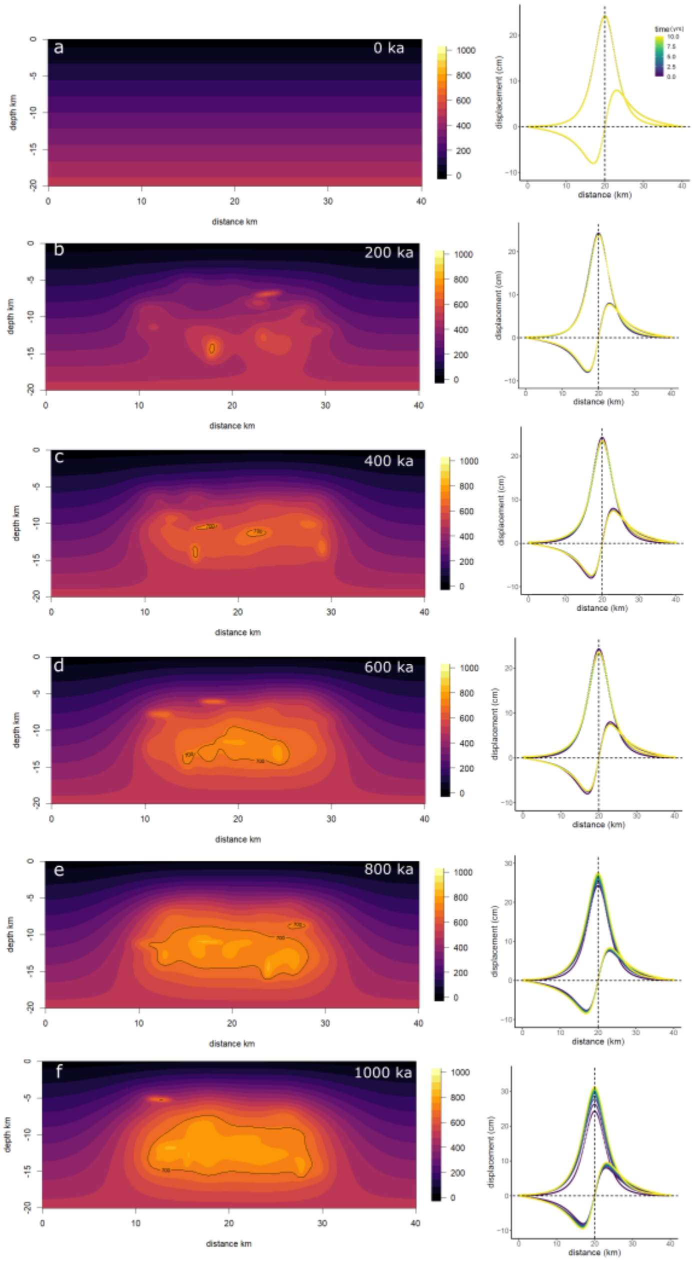

Figure S4. Thermal and deformation modelling results for a larger geometry. The temperature distribution in the crust after different durations of magmatism is shown and the corresponding deformation pattern (horizontal and vertical components) are shown on the right-hand side. All results are for a 1 MPa overpressure source at 5 km depth. Color coding in the deformation plots represents time in years. a) 0 ka of magma injection, b) 200 ka, c) 400 ka, d) 600 ka, e) 800 ka and f) 1000 ka of magma injection.

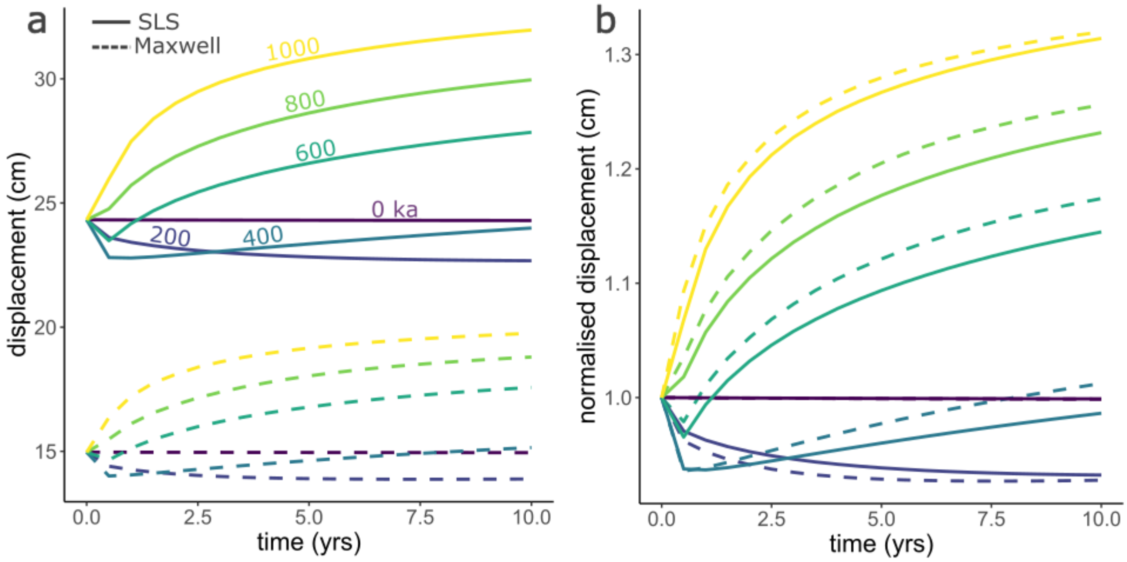

Figure S5. Comparison of Standard Linear Solid (SLS) and Maxwell rheologies. a) Maximum vertical displacement over a 10-year observation period for SLS rheology (solid lines) and Maxwell rheology (dashed). The color coding reflects the duration of magmatism in ka. All results are shown for an overpressure source at 5 km depth. b) Maximum vertical displacement normalized to the initial elastic response.

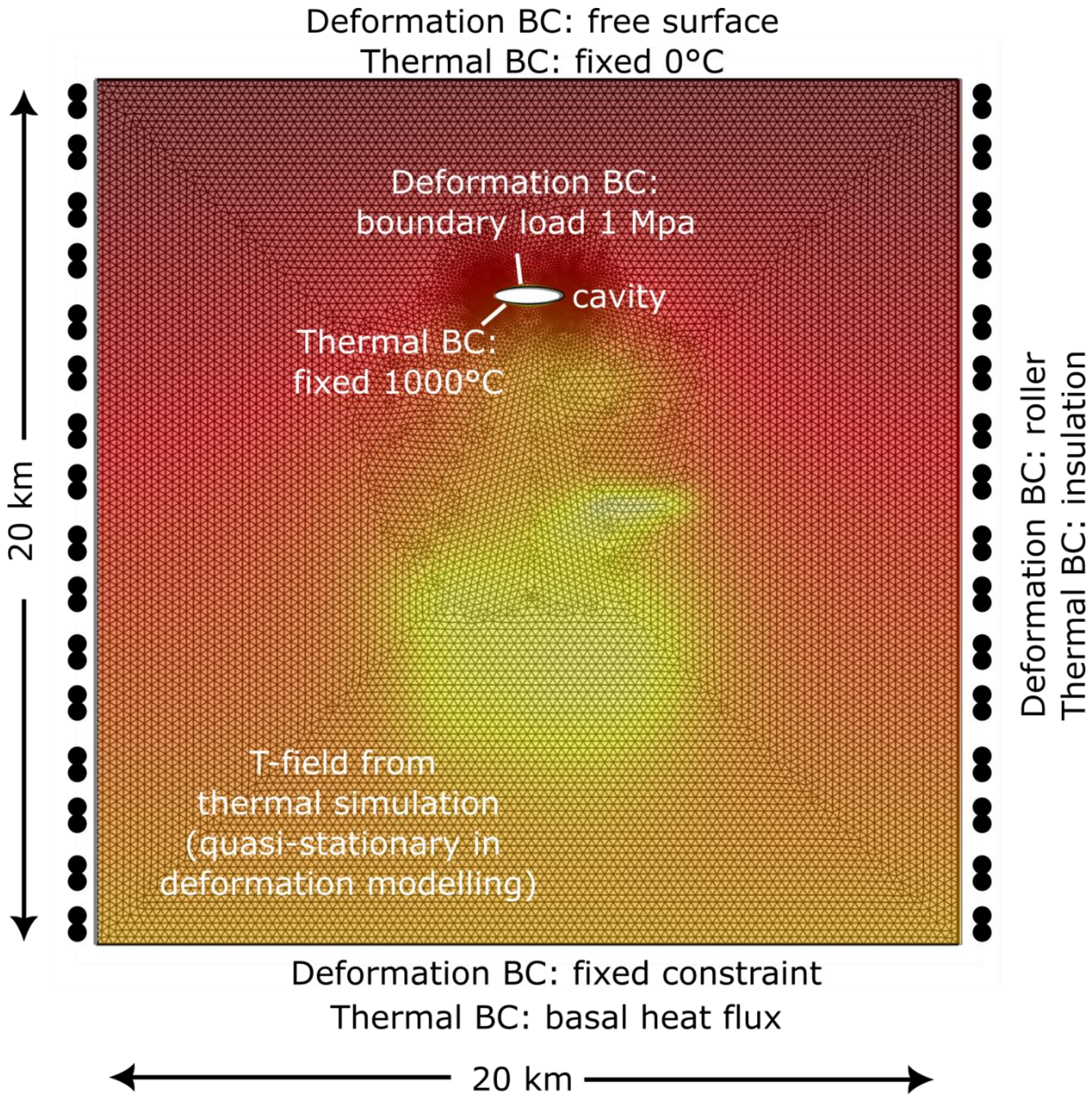

Figure S6. Setup of the finite element thermo-mechanical model. Example of meshing is shown. The background color coding reflects the crustal temperature distribution, which has been implemented from the thermal modelling results. An overpressure source is modelled as an ellipsoidal cavity with boundary load of 1 MPa. BC is for boundary condition.

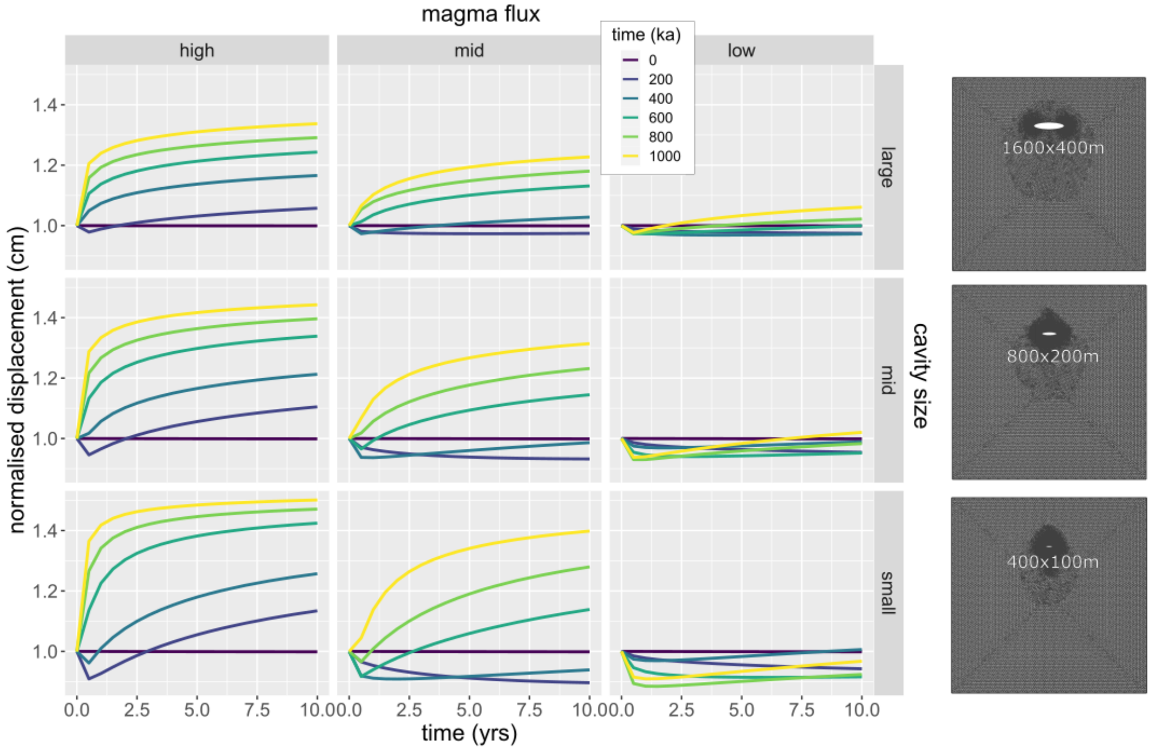

Figure S7. Impact of cavity size. Normalized vertical displacement time series are shown for different magma fluxes (columns: high, mid, low) and as a function of overpressure cavity size (rows: large, mid, small). Color coding reflects the time since the onset of pressurization in ka. The rows show results for large (1600x400m), intermediate (800x200m), and small (400x100m), which are illustrated on the right-hand side.

Table S1: Summary of modelling parameters.

Parameter	Description	value	unit
Tsol	Solidus temperature	700.00	°C
Tliq	Liquidus temperature	1000	°C
dT/dz	Geothermal gradient	25	°C/km
rho	Density (solid and liquid)	2700	kg/m^3
Cp	Heat capacity	1050	J/kg/K
QL	Latent Heat	350000	J/kg
Ad	Dorn parameter	1×10^9	Pas
Ae	Activation energy	120000	J/mol
K	Bulk modulus	13.3×10^9	N/m^2
G	Shear modulus	8×10^9	N/m^2
ΔP	Boundary load	1	MPa

Table S2: Summary statistics of modelled magma reservoir temperature distributions.

model	magma flux	magmatism duration (ka)	Temperature (°C) *Min.*	*1st Qu.* [1]	*Median*	*Mean*	*3rd Qu.*	*Max.*	Deformation pattern
SG_02	high	200	700	719	737	737	748	811	subsidence, uplift
SG_02	high	400	700	742	777	772	799	847	uplift
SG_02	high	600	700	753	794	790	829	872	uplift
SG_02	high	800	700	755	805	799 [2]	845	902	uplift
SG_02	high	1000	700	756	811	804	852	903	uplift
SG_03	mid	200	701	717	739	740 [3]	760	783	subsidence
SG_03	mid	400	700	725	747	748	768	851	subsidence, uplift
SG_03	mid	600	700	731	755	756	781	825	subsidence, uplift
SG_03	mid	800	700	736	772	769	801	833	uplift
SG_03	mid	1000	700	743	779	775	810	879	uplift
SG_03rpt1	mid	200	700	709	726	735	761	796	subsidence
SG_03rpt1	mid	400	700	723	743	742	760	810	subsidence, uplift
SG_03rpt1	mid	600	700	731	751	754	779	815	subsidence, uplift
SG_03rpt1	mid	800	700	738	776	771	804	837	uplift
SG_03rpt1	mid	1000	700	742	776	771	802	839	uplift
SG_05	low	200	-	-	-	-	-	-	subsidence
SG_05	low	400	-	-	-	-	-	-	subsidence, uplift
SG_05	low	600	700	705	715	733	735	758	subsidence, uplift
SG_05	low	800	700	703	706	707	710	720	subsidence, uplift
SG_05	low	1000	700	716	732	733	748	785	subsidence, uplift

For comparision: Temperatures of 725-745°C (Aluto) and 785-905°C (Tullu Moye) have been estimated by Samrock et al. (2021)

Dark grey shaded mean temperatures are within the range of Tullu Moye, light green in the range of Aluto.

[1] Quartile

[2] Simulation shown in Fig. 9f

[3] Simulation shown in Fig. 9e
